# Effect of hyperbaric oxygen therapy (HBO) on osteoblasts of elderly patients on calcification and osteoprotegerin

**DOI:** 10.1016/j.jor.2025.06.017

**Published:** 2025-07-03

**Authors:** Lisa Oezel, Johannes Schneppendahl, Jan-Peter Grassmann, Pia Flender, Sven Dreyer, Vera Grotheer

**Affiliations:** a Department of Orthopedics and Trauma Surgery, Medical Faculty and University Hospital Duesseldorf, Moorenstrasse 5, 40225, Duesseldorf, Germany; b Department of Orthopedics and Trauma Surgery, Evangelisches Krankenhaus Muelheim, Muelheim an der Ruhr, Germany; c Department of Hand- Trauma and Reconstructive Surgery, Am Finkenhügel 1, 49076, Osnabrueck, Germany; d Bielefeld University, Medical School and University Medical Center OWL, Department of Orthopedics Klinikum Bielefeld-Mitte, Germany

**Keywords:** Hyperbaric oxygen therapy (HBO), reactive oxygen species (ROS), Osteoporosis, Calcification, RANK-L, Osteoprotegerin (OPG)

## Abstract

**Background:**

Osteoporosis stands as one of the most prevalent bone diseases worldwide. This study aims to explore the effects of Hyperbaric Oxygen (HBO) therapy and substances that reduce reactive oxygen species (ROS), such as antioxidants, on osteogenic differentiation and key osteoporosis-related parameters in osteoblasts derived from elderly patients requiring hip arthroplasty.

**Methods:**

An in vitro study was conducted using osteoblasts isolated from the femoral heads of 22 patients (78.3 % female) with a mean age of 73 years. Bone mineral density (BMD) was assessed through dual-energy X-ray absorptiometry (DXA), classifying patients into three groups: normal (age-appropriate) BMD (n = 8), osteopenia (n = 6), and osteoporosis (n = 8). Osteogenic differentiation was induced, and HBO therapy was administered over a period of 21 days. Additionally, osteoblasts were treated with catalase. Parameters related to osteogenic differentiation and osteoporosis were evaluated.

**Results:**

HBO therapy prompted osteogenic differentiation in all three experimental groups after 21 days, with statistically significant findings (p = 0.0125) in osteoblasts with age-appropriate bone density. Furthermore, the activity of alkaline phosphatase (ALP), an enzyme indicative of bone synthesis, demonstrated significant increases across all groups following HBO treatment (normal BMD and osteoporotic cells: p = 0.04; osteopenic cells: p = 0.006).

**Conclusions:**

These findings suggest that HBO therapy holds potential as an adjunctive or investigational treatment for elderly patients to enhance bone density or facilitate bone healing post-fracture, especially in those with normal or osteopenic bone density. This approach could potentially influence clinical practices in the future.

## 1 Background

According to the World Health Organization (WHO), osteoporosis is one of the most prevalent bone diseases worldwide, with its incidence rising in older populations.^1^. In 90 % of cases, osteoporosis leads to painful bone fractures, a significant decline in quality of life, and an increased burden on healthcare systems.^2^. Treatment options are limited, with medications such as bisphosphonates being the primary therapy, often leaving patients with multiple side effects and intolerances. For elderly, multimorbid patients, effective therapies are needed to reduce hospital stays and minimize mortality risks.

On a molecular level, osteoporosis pathogenesis is determined by a reduced bone density, caused by heightened osteoclast activity and diminished osteoblast function.^3^. This imbalance is also attributed to a decrease in osteoprotegerin (OPG) expression, resulting in disrupted bone homeostasis.^4^. OPG acts as a decoy receptor that inhibits osteoclast activation by binding to the nuclear factor-κB ligand (RANKL) and preventing its interaction with the RANK receptor on osteoclasts, thereby inhibiting osteoclast activation.^5^. In healthy bone, there is a balance between RANKL and OPG. Disruption in this balance, favoring RANKL, contribute to bone tissue disturbances, as seen in osteoporosis.^6^.

Another important parameter for evaluating osteogenic differentiation is ALP activity. ALP is expressed by osteoblasts during active bone synthesis, and elevated ALP levels are typically observed during osteogenic differentiation, increased bone growth, or fracture healing.^7^. Despite these well-established facts, many aspects of the molecular mechanisms and interactions involved remain unclear.

Reactive oxygen species (ROS) and free radicals, such as hydrogen peroxide (H_2_O_2_), are essential for normal cell function. In bone metabolism, ROS regulate osteoclasts and stimulate bone resorption, having an inhibitory effect on bone metabolism.^8^. However, excessive ROS, particularly in elderly patients, can lead to cellular dysfunction and oxidative stress, a condition linked to the development of various diseases.^9^. Increasing evidence suggests that ROS and oxidative stress play a role in the pathogenesis of bone disorders,^10^, with overexpression of ROS identified in conditions such as diabetic osteoporosis, rheumatoid arthritis, and osteolysis.^11^^,^^12^^,^^13^. Recently, catalase, an antioxidant enzyme known for its ability to degrade large amounts of H_2_O_2_, has been shown to promote OPG expression in osteoblasts from osteoporotic cells and mitigate inhibited osteogenic differentiation.^14^^,^^15^.

Hyperbaric oxygen therapy (HBO) is a technique that allows high concentrations of oxygen to be delivered to body tissues under elevated atmospheric pressure.^16^. HBO is currently used as an adjunctive treatment for a variety of conditions, including open fractures, chronic non-healing wounds, carbon monoxide poisoning, and diving accidents.^17^^,^^18^^,^^19^^,^^20^. HBO has also been reported to positively impact bone regeneration by promoting osteoblast differentiation and improving outcomes for patients with bone diseases.^21^. Specifically, HBO is believed to accelerate osteoblast differentiation while suppressing osteoclastogenesis and activation.^22^^,^^23^, ^25^, ^26^.

Despite these findings, the direct impact of HBO on osteoblasts and bone metabolism especially in the elderly remains underexplored. Therefore, the aim of this study was to investigate for the first time the effects of catalase, a ROS-reducing antioxidant enzyme, and HBO on osteogenic differentiation in osteoblasts with varying bone densities, as well as on key osteoporosis-related parameters such as ALP, OPG, and RANKL.

## 2 Methods

### 2.1 Study design and patient cohort

The study was approved by the institutional review board of our hospital (IRB#: 5585 R), and informed written consent was obtained from all participants. The study adhered to the principles outlined in the Declaration of Helsinki.

### 2.2 Inclusion criteria

Patients over the age of 18 who provided informed written consent were included in the study. Exclusion criteria included patients with chronic or metastatic bone diseases and those unable to undergo postoperative mobility assessments for bone density measurements. A standardized questionnaire was completed by each participant, including demographic information such as age, gender, height, weight, medical history, and current medications.

### 2.3 Eligibility for study group

The study focused on patients requiring hip arthroplasty, either due to an acute femoral neck fracture (classified according to Garden and Pauwels) or due to osteoarthritis with persistent pain and failure of conservative treatments (Kellgren-Lawrence scale ≥ stage II). Written informed consent was mandatory for inclusion in the study.

### 2.4 Exclusion criteria

Exclusion criteria included patients with chronic diseases, carcinogenic conditions, bone metastases, or those unable to participate in postoperative mobility tests for bone density measurements.

### 2.5 Patient demographics

The study cohort consisted of patients with femoral neck fractures or osteoarthritis, from whom osteoblasts were isolated. Patients were grouped based on their bone BMD as determined by dual-energy X-ray absorptiometry (DXA) and classified by their T-scores: the normal BMD group (T-score ≥ −1 g/cm^2^), the osteopenia group (T-score ≥ −1 to −2.5 g/cm^2^), and the osteoporosis group (T-score ≤ −2.5 g/cm^2^). A detailed overview of the patient population is provided in Table 1.

**Table 1:** Patient groups showing the distribution in the three groups (normal, osteopenia, osteoporosis) in relation to their bone status (∅ = mean; ♁ = female patients; ♂︎ = male patients).

BMD Group (n)	Age ∅	Gender (age)	Fracture	Arthrosis
Normal (n = 8)	68.75	6 ♁ (69.3), 2 ♂ (67)	2	6
Osteopenie (n = 6)	81.3	5 ♁ (81.2), 1 ♂ (82)	5	1
Osteoporosis (n = 8)	79.75	6 ♁ (82.5), 2 ♂ (71.5)	7	1

### 2.6 Isolation of human osteoblasts from femoral heads

All cell culture procedures were performed under sterile conditions. Trabecular bone was removed from the femoral heads using a sharp spoon, and the bone fragments were washed with 25 ml of culture medium (DMEM, Pan-Biotech GmbH, Bavaria, Germany). The isolation of osteoblasts followed standard protocols as described in the literature.^24^. The bone fragments were incubated in collagenase type IV solution at 37 °C for 2.5 h on a rotating roller. After incubation, the supernatant was carefully removed, centrifuged at 300 g for 5 min, and the pellet was resuspended in 5 ml of culture medium. This suspension was made up to 50 ml and centrifuged again at 300 g for 5 min.

To confirm the osteoblast identity, osteocalcin immunostaining and morphological observations were performed 3 days after isolation.

### 2.7 Standard cell culture

Osteoblasts were cultured in high-glucose DMEM with 10 % fetal bovine serum (FBS) and 1 % penicillin/streptomycin (Pen/Strep) at 37 °C in a 5 % CO_2_ incubator, maintaining 100 % relative humidity and protection from light. Regular examinations under a light microscope ensured cell vitality, growth, and absence of contamination. The medium was replaced twice weekly. When cells reached 80 % confluence, they were passaged at a 1:3 ratio and distributed into new culture flasks. For osteogenic differentiation, the medium was supplemented with 50 μM L-ascorbic acid-2-phosphate, 10 mM β-glycerophosphate, and 500 nM dexamethasone. All experiments were conducted using the same cell culture passage.

### 2.8 Osteogenic differentiation with and without HBO

Osteoblasts were induced to differentiate using osteogenic differentiation medium (OM), which consisted of standard cultivation medium supplemented with 50 μM L-ascorbate-2-phosphate, 10 mM β-glycerophosphate, 0.1 μM dexamethasone, 125 U/ml catalase, and 50 μM H_2_O_2_. Osteoblasts were seeded in 24-well plates, and once they reached 100 % confluence, they were treated with OM with or without HBO. The HBO treatment was administered 5 times a week for 90 min at 2 bar above sea level, 37 °C, and 100 % oxygen. The medium was replaced twice a week. Osteogenic differentiation was assessed on days 0 and 21 using the alizarin red S staining. For each time point, the stainings were performed in sextuplet, and the day 0 value was subtracted.

### 2.9 The effect of HBO on pH value

To determine if HBO therapy affected the pH value of the medium, a control experiment was conducted. Osteoblasts from one donor were used to investigate pH changes after HBO treatment. The osteogenic medium (OM) was treated with HBO for three consecutive days, as described above, and the pH value was measured using a pH meter (HI2020 edge). The pH value of the HBO-treated medium was found to be 8.4, which prompted the preparation of an alkaline OM solution to match this pH. Osteoblasts were then treated with OM ± catalase for 21 days, and a third group received the alkaline OM solution (pH = 8.4). The alizarin red S staining was conducted on day 21.

### 2.10 Alizarin red S staining

In order to qualify and quantify the osteogenic differentiation potential of osteoblasts and their ability to undergo matrix mineralization, alizarin red S staining was used. Terminally differentiated osteoblasts (osteocytes), as well as active osteoblasts form extracellular matrix proteins (collagen, decorin, osteocalcin) into which calcium phosphate was incorporated. Alizarin red S stains those calcium ions. Alizarin red S is a dye that binds selectively to calcium salts and is widely used for calcium mineral histochemistry. Adherent osteoblasts monolayers were washed with PBS and fixed with 4 % paraformaldehyde for 15 min, rinsed 2 times with PBS, covered for 20 min at 37 °C with alizarin red S solution (0.5 % in aqua dest., pH = 4.1) and washed with dH_2_O until the supernatant was colorless. Stained monolayers were visualized by phase microscopy using an inverted microscope (Zeiss Axiovert 200 microscope). This process was followed by a quantitative destaining procedure using 10 % (w/v) cetylpyridinium chloride in 10 mM sodium phosphate, pH = 7.0, for 25 min at room temperature. The alizarin red S concentration was determined by absorbance measurement at **λ** = 600 nm. For each time point, a sextuple determination was performed.

### 2.11 ALP activity measurement

ALP activity was measured on days 0 and 21. Osteoblasts were incubated with 10 mM 4-nitrophenol solution for 15 min. The absorbance changes were quantified using a photometer at **λ** = 405 nm (PerkinElmer Victor 2 plate reader). Day 0 values were subtracted from day 21, and duplicate analyses were performed. Absorbance values were normalized to sample viability.

### 2.12 Cell viability assay

Cell viability was assessed using the CellTiter-Blue Assay (Promega, Madison, USA) after ALP activity measurement. The working solution was prepared by diluting CellTiter-Blue in medium (1:20). After incubating for 1 h, fluorescence (540Ex/590 Em) was measured in a 1420 Multilabel Counter (Victor3, PerkinElmer).

### 2.13 OPG-ELISA

Osteoblast supernatants were diluted (1:3) and 100 μl of the samples were added in duplicate to ELISA wells following the manufacturer's instructions (DuoSet ELISA, R&D Systems, DY805). Optical density was measured using a Victor X3 Multilabel Reader (PerkinElmer).

### 2.14 RANKL mRNA expression analysis

RNA was extracted from osteoblasts using the RNeasy Mini Kit according to the manufacturer's specifications (Qiagen, Hilden, Germany). One microgram of RNA was reverse-transcribed into cDNA using the QuantiTect Reverse Transcription Kit (Qiagen), with an extended incubation time of 30 min at 42 °C. Quantitative reverse transcription polymerase chain reaction (qRT-PCR) was performed with PowerUp™ SYBR® Green Master Mix (Applied Biosystems®, Dreieich, Germany) according to the manufacturer's instructions on the StepOne Real-Time PCR System (Applied Biosystems®, Dreieich, Germany). RNA expression was measured using the RANKL primers (Hs_TNFSF11_1_SG, Sequenz: QT00215614) and as housekeeping gene, glyceraldehyde-3-phosphate-dehydrogenase (GAPDH) was used. GAPDH (h-GAPDH-Reverse Sequence: 5′-CTC CTG GAA GAT GGT GAT GG-3′, h-GAPDH-Forward Sequence: 5′-ACG GAT TTG GTC GTA TTG GGC G-3) qRT-PCR was performed using initial denaturation at 95 °C for 2 min and 45 cycles of amplification, including denaturation at 95 °C for 10 s, annealing and elongation for 30 s at 60 °C, and a melting curve analysis.^27^.

Timepoints for OPG and RANKL analysis were chosen to be on days 14 and day 21 due to the results of the working group led by Huang et al., 2004, who were able to observe a peak expression of OPG and RANKL on day 14.^28^.

### 2.15 Statistical analysis

Data were analyzed using GraphPad Prism (Version 8.1.2, San Diego, CA, USA). Normality was assessed using the D'Agostino-Pearson test. For normally distributed data, a two-way ANOVA was used to evaluate the effect of therapy across different variables (same donor but different cells). A p-value ≤0.05 was considered.

## 3 Results

A total of twenty-two patients (17 women, 5 men) participated in this study. The normal BMD group consisted of eight donors (6 women, average age 69.83 years; 2 men, average age 67 years) with age-appropriate bone density (T-score ≥ −1 g/cm^2^). The osteopenia group included six donors (5 women, average age 81.2 years; 1man, average age 82 years) with reduced bone density (T-score between ≥ −1 and −2.5 g/cm^2^). Lastly, the osteoporosis group comprised eight donors (6 women, average age 81.67 years; 2 men, average age 74 years) with significantly decreased bone density (T-score ≤ −2.5 g/cm^2^) (see Table 1) A more detailed table including gender, height, weight and medical history of the patients can be found in Supplement 1C.

### 3.1 Effect of HBO on pH value

Our experiments revealed that HBO treatment increased the pH value of osteogenic medium (OM) to 8.4. However, when an alkaline OM with a pH of 8.4 was used without HBO, osteogenic differentiation of osteoblasts was reduced (Supplement 1B). This outcome suggests that the beneficial effects of HBO on osteogenic differentiation are not attributable to the pH increase but rather to the specific influence of HBO therapy itself.

### 3.2 Effect of HBO and catalase on osteogenic differentiation of osteoblasts as assessed by alizarin red S staining

Osteogenic differentiation was observed in all three experimental groups after 21 days, with both HBO and the combination of HBO and catalase enhancing differentiation (Fig. 1.1).

**Fig. 1:**
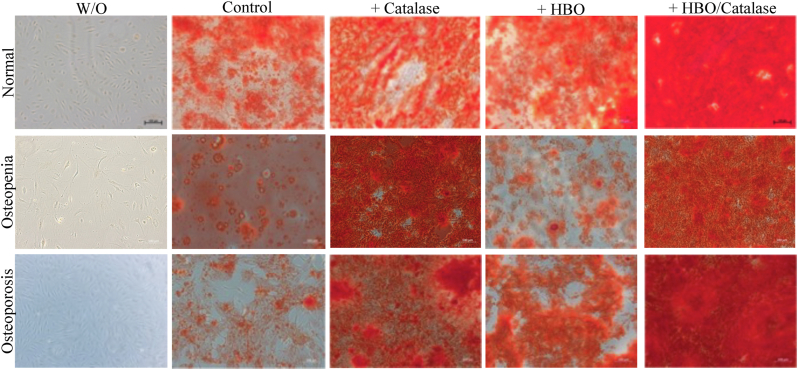

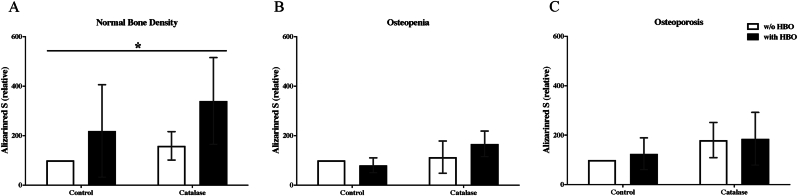
Effect of HBO and catalase on osteogenic differentiation of ostoeoblasts Fig. 1.1 Representative images of the osteogenic differentiation of osteoblasts visualized with alizarin red S staining after 21 days. The measuring bar represents a length of 100 µm. Rows are as stated in the image: Row 1: normal BMD, Row 2: osteopenic BMD, Row 3: osteoporotic BMD. From left, Column 1: no additives, Column 2: osteogenic diff. medium, Column 3: osteogenic diff. medium + catalase, Column 4: osteogenic diff. medium + HBO and Column 5: osteogenic diff. medium + catalase + HBO. Below: Effect of HBO on osteogenic differentiation in osteoblasts from elderly patients. Matrix mineralization was analyzed with alizarin red S staining and quantified. Fig 1.2 A: In osteoblasts from patients with normal bone mineral density HBO therapy significantly improved osteogenic differentiation potential (n = 8); * = p < 0.05; Fig 1.2 B: In osteoblasts from patient with osteopenic bone mineral density (n = 6); Fig 1.2 C: Osteoblasts with osteoporotic bone mineral density (n = 8). Black bars represent HBO treatment and white bars the respective controls.

In the normal BMD group, treatment with HBO resulted in a significant increase in osteogenic differentiation, with values nearly twice as high as those of the untreated group (p = 0.0125) (Fig. 1.2A).

In the osteopenia group (Fig. 1.2B), HBO treatment caused a slight decrease in osteogenic differentiation, whereas the combination of HBO and catalase led to a more pronounced increase. However, neither result was statistically significant.

In the osteoporosis group (Fig. 1.2C), both HBO and the HBO + catalase combination promoted osteogenic differentiation. Notably, the combined treatment of HBO + catalase appeared more effective than HBO alone.

### 3.3 Determination of ALP in osteoblasts after HBO and catalase treatment

**Fig. 2:**
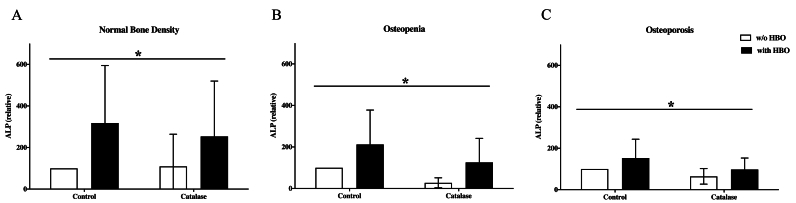
Effect of HBO on ALP activity in osteoblasts from elderly patients. A: In osteoblasts with normal bone mineral density HBO significantly improved ALP activity (n = 8); B: In osteoblasts with osteopenic bone mineral density HBO significantly improved ALP activity (n = 6); C: In osteoblasts from patients with osteoporotic bone mineral density HBO significantly elevated ALP activity (n = 8). ∗ = p < 0.05.

In the group of osteoblasts with age-appropriate bone density, a significantly higher ALP activity of nearly two-to threefold could be detected with HBO therapy compared to the treatment without HBO therapy (p = 0.04) (Fig. 2A).

In the osteopenia group, there was a significant increase in ALP activity induced by HBO of almost threefold compared to the treatment without HBO (p = 0.006) (Fig. 2B).

In osteoblasts with osteoporotic bone density, HBO significantly increased ALP (p = 0.04) (Fig. 2C). Collectively, treatment of osteoblasts with catalase decreased ALP activity in all groups, although not significantly.

### 3.4 OPG expression in osteoblasts after HBO and catalase treatment

**Fig. 3:**
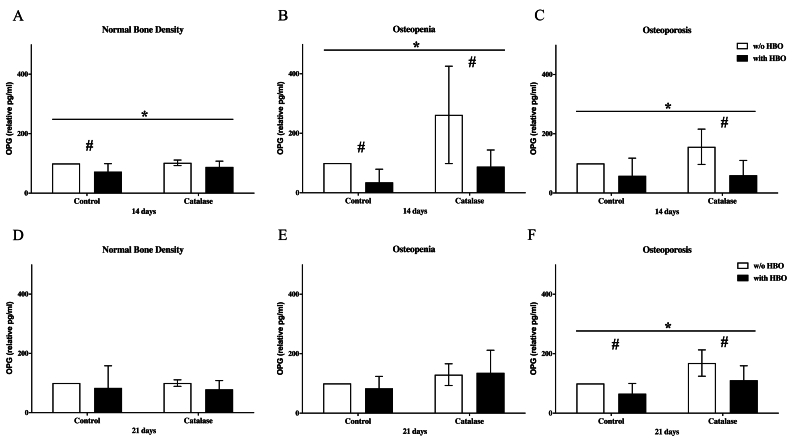
The impact of HBO and catalase treatment on osteoblast's OPG secretion **A**: The HBO significantly decreased OPG expression after 14 days treatment in osteoblasts with normal bone mineral density. In osteoblasts control group (treatment w/o catalase) this effect was significant, too (n = 8). **B**: The HBO significantly decreased OPG expression after 14 days treatment in osteoblasts with osteopenic bone mineral density. But the expression also differed significantly within the groups (with an w/o catalase) (n = 6); **C**: The HBO significantly decreased OPG expression after 14 days treatment in osteoblasts with osteoporotic bone mineral density. Here the catalase treatment is also significantly improved to their respective control (n = 8). # = p < 0.05 (significance between two groups analyzed by *t*-test); ∗ = p < 0.05 (significance within all groups analyzed by two-way ANOVA test) **D**: The HBO had no relevant effect on OPG expression after 21 days treatment in osteoblasts with normal bone mineral density; (n = 8). **E:** The HBO had no relevant effect on OPG expression after 21 days treatment in osteoblasts with osteopenic bone mineral density (n = 6); **F:** The HBO significantly decreased OPG expression after 21 days treatment in osteoblasts with osteoporotic bone mineral density. Here both groups with and w/o catalase treatment differed also significantly compared to their respective controls (n = 8). # = p < 0.05 (significance between two groups analyzed by *t*-test); ∗ = p < 0.05 (significance within all groups analyzed by two-way ANOVA test).

In osteoblasts with normal bone density, HBO therapy resulted in a significant reduction in OPG expression after 14 days (p = 0.002) (Fig. 3). Similarly, osteoblasts from patients with osteopenic bone density exhibited a notable decrease in OPG levels following HBO treatment (p = 0.004) (Fig. 3B). In osteoporotic osteoblasts, OPG secretion was also significantly suppressed by HBO therapy (p = 0.0008), aligning with the previously observed trends. However, as seen in osteopenic osteoblasts, an increase in OPG concentration was observed when treated with both HBO and catalase (Fig. 3C). After 21 days, no significant modulation of OPG expression was observed in osteoblasts with normal bone density following treatment with either HBO or catalase (Fig. 3D). In osteoblasts from patients with osteopenic bone density, OPG levels became comparable regardless of HBO treatment, showing no significant differences from the measurements at day 14 (Fig. 3E).

In contrast, osteoblasts from patients with osteoporosis demonstrated a significant reduction in OPG expression (p = 0.002) after 21 days of HBO treatment (Fig. 3F). Furthermore, the combined application of HBO and catalase resulted in a significant increase in OPG expression (p < 0.0001).

### 3.5 Determination of RANKL expression in osteoblasts after HBO and catalase treatment

**Fig. 4:**
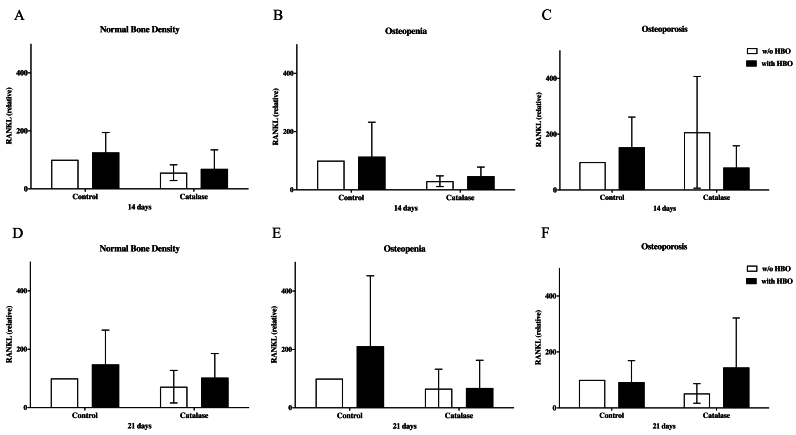
Effect of HBO and catalase on RANKL expression in osteoblasts from elderly patients. A: In osteoblasts with normal BMD RANKL expression was slightly increased, but this effect was not significant (n = 8); B: The HBO had no relevant effect on RANKL expression after 14 days treatment in osteoblasts with osteopenic bone mineral density (n = 6); C: The HBO had no relevant effect on RANKL expression in osteoblasts with osteoporotic bone mineral density (n = 8). D: In osteoblasts with normal BMD RANKL expression was slightly increased after 21 days, but this effect was not significant (n = 8); E: The HBO had no relevant effect on RANKL expression after 21 days treatment in osteoblasts with osteopenic bone mineral density (n = 6); F: The HBO had no relevant effect on RANKL expression after 21 days treatment in osteoblasts with osteoporotic bone mineral density (n = 8).

HBO did not significantly affect the regulation of RANKL in osteoblasts with age-appropriate BMD after 14 days (Fig. 4A). Similarly, no notable modulation of RANKL was observed in osteopenic osteoblasts following HBO treatment at day 14 (Fig. 4B). However, the addition of catalase appeared to slightly increase RANKL expression in osteopenic osteoblasts, although this change was not statistically significant. In osteoporotic osteoblasts, HBO treatment led to an increase in RANKL production after 14 days, but the results were not significant. Interestingly, the combined treatment of HBO and catalase resulted in a decrease in RANKL concentration (Fig. 4C). After 21 days, HBO therapy did not lead to any significant increase in RANKL expression in osteoblasts with normal bone density (Fig. 4D). Similarly, in osteopenic osteoblasts, no significant modulation of RANKL expression by HBO was observed at day 21 (Fig. 4E). In osteoporotic osteoblasts, there was a tendency toward increased RANKL expression following HBO treatment when compared to the measurements taken at day 14 (Fig. 4F).

## 4 Discussion

In the present study, we investigated the impact of HBO on osteogenesis and osteoporosis-related parameters, such as ALP, OPG, and RANKL, in osteoblasts derived from elderly patients.

Our findings revealed that HBO therapy induced osteogenic differentiation across all three experimental groups (normal BMD, osteopenia, and osteoporosis) after 21 days. Furthermore, ALP activity exhibited significant increases in all groups following HBO treatment (normal BMD and osteoporotic cells: p = 0.04; osteopenic cells: p = 0.006). OPG levels were reduced by HBO therapy, but the addition of catalase to HBO resulted in an increase. Similarly, RANKL levels were elevated when osteoblasts were treated with both HBO and catalase.

Oxygen has been suggested to play a regulatory role in bone remodeling, notably by directly influencing ALP activity and transforming growth factor-β (TGF-β) production at fracture sites.^29^,^30^. Previous studies have also indicated that elevated oxygen levels, such as those provided by HBO, can enhance osteogenic differentiation.^31^. In experiments conducted by Wu et al., osteoblasts treated with HBO for 90 min at 2.4 ATA exhibited a significantly higher cell count and increased calcium incorporation into the extracellular matrix (ECM) compared to control groups not receiving HBO treatment.^32^. These results align with our own, which demonstrate that osteoblasts exposed to 100 % pure oxygen at 2 bar pressure also exhibited enhanced osteogenic differentiation, as evidenced by increased calcium incorporation into the ECM.

The literature offers limited information regarding the specific oxygen concentration and HBO pressure parameters. Wu et al. proposed that only HBO pressure, rather than oxygen concentration, is responsible for osteogenic differentiation.^32^. In contrast, two other studies suggest that oxygen concentration plays a critical role, with 5 % oxygen being insufficient and promoting the formation of a cartilaginous matrix, while a higher oxygen concentration (at least 35 %) is necessary for tissue differentiation into bone. The influence of varying oxygen levels on osteogenic differentiation in osteoblasts from elderly patients remains debatable.^33^. Furthermore, Monfoulet et al. suggested that increased HBO pressure (2 bar above sea level) can lead to alkalization of the differentiation medium, inhibiting osteogenic differentiation.^34^. In our study, however, the HBO pressure and concentration used (2.0 ATA and 100 % O_2_) align with those in previous literature, where similar conditions have resulted in positive outcomes, such as reduced inflammation, improved vascular perfusion, and enhanced tissue regeneration.^35^.

The influence of pH on osteogenic differentiation has also been explored. Monfoulet et al. demonstrated that osteogenesis and mineralization of bone structures derived from human mesenchymal stromal cells (hBMSCs) were strongly dependent on the pH of the cell culture medium. Alkaline pH values above 7.9 significantly impaired osteogenic differentiation, with complete inhibition occurring at a pH of 8. Their findings suggested that osteogenesis decreased 20 - 30-fold when the pH increased from 7.5 to 7.8, with no impact on proliferation within the pH range of 7.9–8.27. In contrast, our study showed that HBO therapy increased the pH value, yet osteoblasts still exhibited positive osteogenic differentiation behavior (see Supplements 1B). Furthermore, our additional experiment using alkaline osteogenic medium suggested that the positive effect of HBO on osteogenesis was not primarily due to changes in pH, but rather the HBO effect itself.

The composition of the differentiation medium warrants consideration. In our study, HEPES was added as a buffer to the osteogenic medium to minimize pH fluctuations. Monfoulet et al. also reported that HEPES acted as a buffering agent, promoting the maintenance of an optimal pH level for osteogenic differentiation (see Fig. 2).

Our results demonstrated that HBO significantly increased ALP activity, consistent with findings in the literature that show HBO treatment leads to elevated ALP concentrations in osteoblasts.^32^. Moreover, calcium storage in the extracellular matrix (ECM) was also increased by HBO treatment, confirming previous reports.^36^. Specifically, osteoblasts from individuals with normal BMD exhibited a 2- to 3-fold increase in ALP activity following HBO treatment compared to untreated cells. Osteoblasts from osteopenic (p = 0.0006) and osteoporotic (p = 0.004) patients also showed significant increases in ALP activity with HBO therapy.

RANKL and RANK play crucial roles in bone metabolism. Overactivation of the RANKL/RANK signaling pathway leads to an imbalance in bone remodeling, favoring resorption.^37^. Interestingly, the effect of HBO combined with catalase on RANKL expression varied between 14 and 21 days (see Fig. 4). This could be explained by the fact that osteoblasts from aged donors were cultured in osteogenic differentiation medium for 21 days, and the secretion of RANKL may follow a cyclical pattern, as observed for ALP expression or Wnt signaling regulation. Additionally, RANKL secretion tends to decrease with prolonged cultivation in osteogenic medium, as demonstrated by Chen et al.^38^ and Jeong et al..^39^.

OPG serves as a decoy receptor for RANKL, inhibiting RANKL from binding to RANK and thereby reducing bone resorption. This signaling pathway and its pathomechanism are relevant in both osteonecrosis and osteoporosis. We compared our results with the few existing studies on osteonecrosis and HBO, particularly focusing on the RANKL/RANK/OPG pathway. Vezzani et al. observed a significant increase in soluble OPG following HBO treatment in patients with avascular necrosis of the femoral head.^16^. Bosco et al. demonstrated that HBO reduced inflammatory cytokines (TNF-α, IL-1β, IL-6) in patients with avascular bone necrosis.^40^, ^41^. Additionally, HBO therapy promotes healing of necrotic tissue by stimulating angiogenesis, fibroblast and osteoblast proliferation, and collagen formation, primarily through ROS-mediated signaling pathways.^40^,^42^. In our study, we found that HBO therapy significantly decreased OPG expression, which was subsequently increased with the addition of catalase. One possible explanation is that ROS levels disrupt osteoblast function, reducing OPG expression and increasing osteoclast activation. Catalase, by breaking down ROS, helped restore OPG levels.

The role of ROS in bone metabolism has been partially explored. An et al. demonstrated that oxidative stress causes bone cell damage and disrupts bone remodeling. Antioxidants are essential for preventing and treating osteoporosis by scavenging ROS and reducing oxidative stress. The authors recommended combining antioxidants with other osteoporosis treatments (e. g., bisphosphonates) or using them as a sole therapy for osteoporosis.^43^. Zhou et al. discussed the potential therapeutic effects of antioxidants (vitamins C and E, selenium, phenols) in osteoporosis, suggesting they may reduce fracture risk by protecting bone metabolism.^14^^,^^44^. Catalase and superoxide dismutase (SOD) are key enzymes in reducing oxidative stress, and catalase, due to its high turnover rate, is particularly effective for detecting and mitigating oxidative damage.^45^^,^^46^. In our study, catalase was analyzed as an antioxidant enzyme, demonstrating its potential to enhance osteogenic differentiation in conjunction with HBO therapy. The findings suggest that antioxidants may positively influence bone metabolism, especially in osteopenic or osteoporotic conditions.

In conclusion, while HBO therapy showed promise in improving osteogenic differentiation across all patient groups (normal BMD, osteopenia, osteoporosis), catalase treatment exhibited even more significant positive effects on osteogenic parameters. These results underscore the potential of antioxidants in modulating bone metabolism. However, further research is required to fully understand the benefits and risks of antioxidants in osteoporosis therapy, and whether ROS modulation through antioxidants could become a complementary treatment for patients with osteoporosis.

Moreover, HBO therapy might provide additional benefits for osteoporotic patients, such as promoting fracture healing. However, further studies with larger sample sizes and deeper analyses are necessary to better understand these effects. Given the high cost and limited availability of HBO chambers, more research is needed to determine whether HBO could offer a cost-effective alternative to existing therapies, such as anti-resorptive drugs or monoclonal antibodies, which are financially burdensome for healthcare systems.

## 5 Limitations

Our study has several limitations. First, osteoblast samples were collected over four years, with a limited sample size of 22 patients due to surgical constraints at our hospital. This sample size is a common limitation in Level II studies. Second, the experiments were performed ex vivo, limiting the translation of results to clinical practice. Additionally, the nature of the study introduces potential biases, such as the exclusion of patients with chronic or carcinogenic bone diseases, and other comorbidities or medications that could have influenced the outcomes. Furthermore, the study does not provide long-term data on HBO therapy, which could reveal potential side effects or disadvantages. Finally, as the osteoblasts were sourced from elderly patients, further age-related changes in cell behavior, such as proliferation and protein expression, must also be considered.

Despite these limitations, our study offers valuable initial insights into the effects of HBO on osteogenic differentiation. Future studies with larger sample sizes, longer follow-up periods, and investigations into optimal dosing and duration of HBO and catalase therapy are essential for better understanding the in vivo implications of our findings and their clinical applications.

## 6 Conclusions

HBO combined with catalase, an antioxidant enzyme, can enhance osteogenic differentiation in human osteoblasts from elderly patients, particularly those with normal BMD. Patients with established osteoporosis may benefit more from antioxidant supplementation. These findings could contribute to the development of methods for accelerating fracture healing or optimizing bone-implant integration after arthroplasty. Furthermore, additional research is necessary to investigate HBO and catalase as potential preventive treatments for bone strengthening through enhanced osteogenic differentiation in pre-damaged bone. Prospective clinical trials assessing functional, radiographic, and serological outcomes in patients with osteopenia or osteoporosis undergoing HBO would provide the most valuable insights.

## Informed consent for publication

Informed written consent was obtained from all participants. The authors certify that they have obtained all appropriate patient consent forms. In the form the patient(s) has/have given his/her/their clinical information to be reported in the journal. The patients understand that their names and initials will not be published and due efforts will be made to conceal their identity, but anonymity cannot be guaranteed.

## Ethics approval and consent to participate

The study protocol was approved by our hospital's institutional review board (IRB#:5585 R). Informed written consent was obtained from all participants, and the study was conducted according to the principles expressed in the Declaration of Helsinki.

## Authors contributions

LO: conceived and designed study and analysis, collected data, manuscript drafting, JS: conceived and designed study and analysis, collected data, manuscript drafting, JPG: collected the data, performed analysis, PF: collected data, SD: conceived and designed study, JW: study supervision, VG: conceived and designed study, study supervision.

## Funding/sponsorship

This research received funding by the GTÜM (Gesellschaft für Tauch-und Überdruckmedizin).

## Conflict of interest

None.
