# Supplementary material for: Effect of hyperbaric oxygen therapy (HBO) on osteoblasts of elderly patients on calcification and osteoprotegerin
Source: J Orthop. 2025 Jul 3;67:335–43. doi: 10.1016/j.jor.2025.06.017 (PMC12304694; doi:10.1016/j.jor.2025.06.017)
Supplement: Multimedia component 1 [file mmc1.pdf]

A

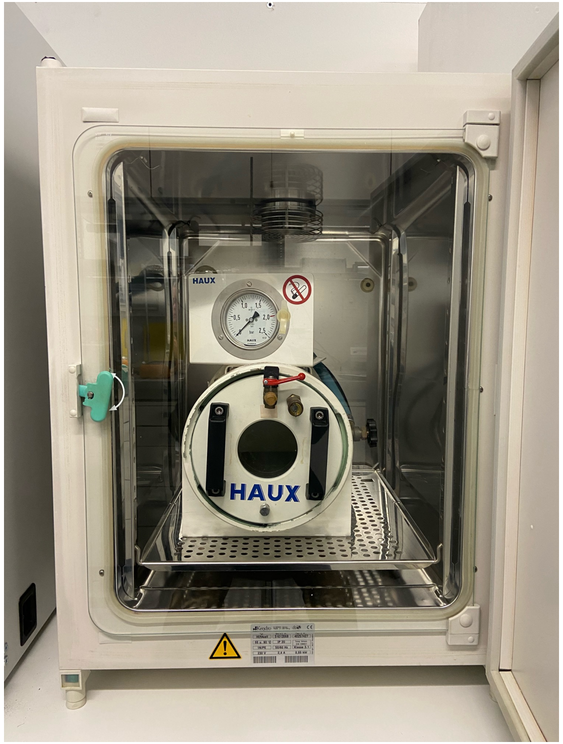

Supplement 1A: HBO HAUX Laboratory pressure chamber

B

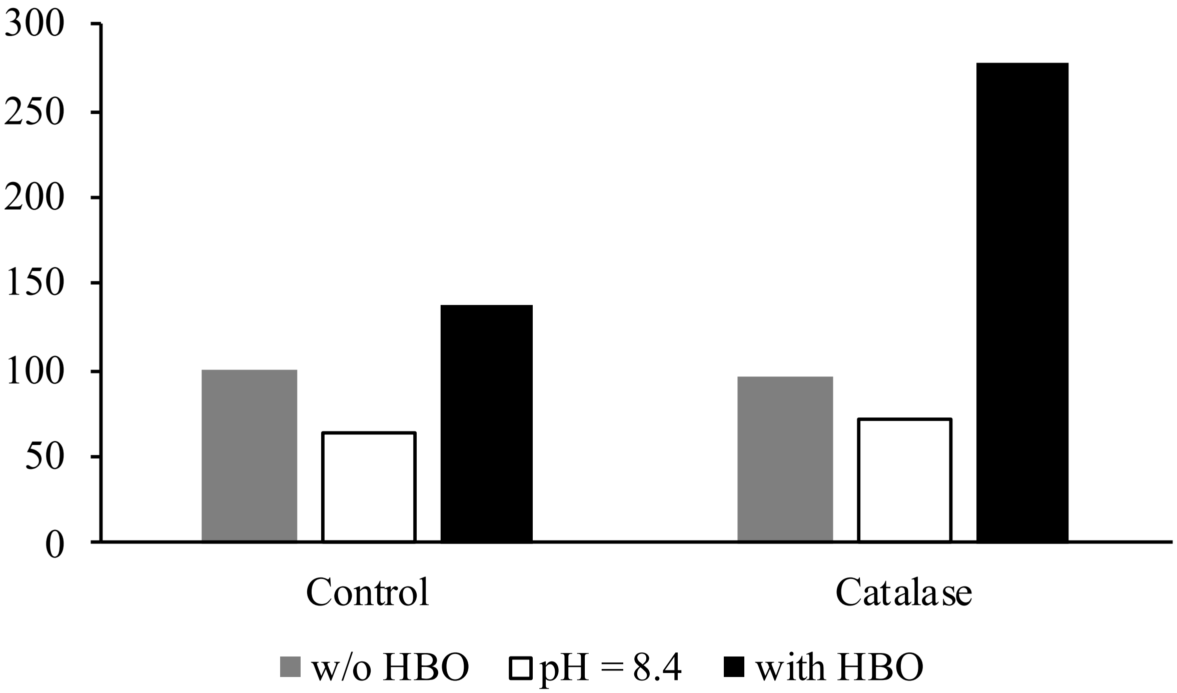

Supplement 1B: Analysis of pH value on osteogenic differentiaiton evalutated with alizarin red s. The use of an alkaline medium decreased osteogenic differentiation potential in osteoblasts.
